# Oxygen uptake ( $\dot{V}$ O_2_) and pulmonary ventilation ( $\dot{V}$ E) during military surface fin swimming in a swimming flume: Effects of surface immersion

**DOI:** 10.3389/fphys.2023.1145204

**Published:** 2023-03-06

**Authors:** Olivier Castagna, Jean-Eric Blatteau, Arnaud Druelle, Jordan Amara, Jean-René Lacour

**Affiliations:** ^1^ Underwater research team—ERRSO, Military biomedical research institute-IRBA, Toulon, France; ^2^ LAMHESS (UPR 6312), Université de Nice, Nice, France; ^3^ Department of underwater and hyperbaric medicine (SMHEP), Ste Anne military hospital (HIA Ste Anne), Toulon, France; ^4^ French Navy Diving school, St Mandrier, France; ^5^ Military Diver Unit, Brest, France; ^6^ Université Jean Monnet, Ste Etienne, France

**Keywords:** fin swimming, oxygen uptake, pulmonary ventilation, heart rate, lactate, swim flume

## Abstract

**Introduction**: During military fin swimming, we suspected that oxygen uptake ( $\dot{V}$ O_2_) and pulmonary ventilation ( $\dot{V}$ E) might be much higher than expected. In this framework, we compared these variables in the responses of trained military divers during land cycling and snorkeling exercises.

**Methods**: Eighteen male military divers (32.3 ± 4.2 years; 178.0 ± 5.0 cm; 76.4 ± 3.4 kg; 24.1 ± 2.1 kg m^-2^) participated in this study. They performed two test exercises on two separate days: a maximal incremental cycle test (*land* condition), and an incremental fin swimming (*fin* condition) in a motorized swimming flume.

**Results**: The respective *fin* and *land* $\dot{V}$ O_2max_ were 3,701 ± 39 mL min^-1^ and 4,029 ± 63 mL min^-1^ (*p* = 0.07), these values were strongly correlated (*r* ^2^ = 0.78 *p* < 0.01). Differences in $\dot{V}$ O_2*max*_ between conditions increased relative to *l;* $\dot{V}$ O_2max_ (*r* ^2^ = 0.4 *p* = 0.01). *Fin* $\dot{V}$ E_*max*_ values were significantly lower than *land* $\dot{V}$ E_*max*_ values (*p* = 0.01). This result was related to both the significantly lower *fin* Vt and *f* (*p* < 0.01 and <0.04, respectively). Consequently, the *fin* $\dot{V}$ E_*max*_/ $\dot{V}$ O_2*max*_ ratios were significantly lower than the corresponding ratios for *land* values (*p* < 0.01), and the *fin* and *land* $\dot{V}$ E_*max*_ were not correlated. Other parameters measured at exhaustion—PaO_2_, PaCO_2_, and SO_2_ - were similar in *fin* and *land* conditions. Furthermore, no significant differences between *land* and *fin* conditions were observed for peak values for heart rate, blood lactate concentration, and respiratory exchange ratio R.

**Conclusion:** Surface immersion did not significantly reduce the $\dot{V}$ O_2*max*_ in trained divers relative to *land* conditions. As long as $\dot{V}$ O_2_ remained below $\dot{V}$ O_2*max*_, the $\dot{V}$ E values were identical in the two conditions. Only at $\dot{V}$ O_2*max*_ was $\dot{V}$ E higher on land. Although reduced by immersion, $\dot{V}$ E_*max*_ provided adequate pulmonary gas exchange during maximal fin swimming.

## Introduction

During immersed physical exercise, cardiovascular, ventilatory and metabolic capacities of divers are altered by at least three mechanisms: the specific thoraco-pulmonary effects of immersion (Morrison and Butt, 1972; Morrison et al., 1975; Prefaut et al., 1976; Taylor and Morrison, 1999; Taylor et al., 2014; Castagna et al., 2018b; Castagna et al., 2021); the effects related to the breathing apparatus (Moon et al., 2009); and the effects of the depth of immersion which increases the density and thus the viscosity of the gases breathed (Kao, 1963; Lanphier, 1963; Davies et al., 1970; Morrison and Butt, 1972; Morrison et al., 1975; Morrison et al., 1976; Thalmann et al., 1979; Warkander et al., 1992).

Studies of oxygen uptake underwater have been undertaken with subjects counteracting active drag (Di Prampero et al., 1974), or swimming on a swimming flume since this device was first described by Holmér and Åstrand (Holmer and Astrand, 1972). Several groups have described an increase in $\dot{V}$ O_2_ in response to increased swimming speed or thrust force (Donald and Davidson, 1954), (Goff et al., 1957), (Pendergast and Lundgren, 2009), (Wylegala J. et al., 2007), (Yamaguchi et al., 1995). Zamparo et al. (Zamparo et al., 2006) calculated the work developed by fin swimmers as a function of speed. Interestingly, in both Yamaguchi et al. (Yamaguchi et al., 1995) and Wylegala et al. (Wylegala J. A. et al., 2007), $\dot{V}$ E, and $\dot{V}$ O_2_ were reported to plateau at the highest work rates. However, compared to cycling, Jammes et al. (Jammes et al., 2009) reported $\dot{V}$ E_max_ to be decreased by 36%.

Military surface fin diving is an intense activity that can result in accidents. This activity thus deserves thorough investigation. Immersion pulmonary edemas (IPE) can develop during fin swimming exercises (with a snorkel or a diving breathing apparatus). Indeed, IPE are the first cause of hospitalization among military divers, more frequent even than decompression accidents (Coulange et al., 2010; Gempp et al., 2011; Castagna et al., 2017; Castagna et al., 2018a). It is now well established that the increased ventilatory and cardiovascular demands induced by fin swimming contribute to the occurrence of IPE (Fraser et al., 2011; Peacher et al., 2015; Moon et al., 2016; Moon, 2019; Wilmshurst, 2021; Hageman et al., 2022). We must therefore precisely measure these adaptations to ensure that military divers are adequately trained to withstand the constraints associated with their diving in practice.

The first purpose of this study was to check whether fin swimming altered maximal pulmonary ventilation ( $\dot{V}$ E_*max*_), as reported elsewhere (Yamaguchi et al., 1995), (Wylegala J. et al., 2007), (Jammes et al., 2009). If this effect was confirmed, we aimed to check whether ventilation was limiting during a surface snorkeling exercise.

## Methods

### Subjects

Eighteen male divers (32.3 ± 4.2 yrs; 178.0 ± 5.0 cm; 76.4 ± 3.4 kg; 24.1 ± 2.1 kg m^-2^) participated in this study. Subjects were healthy non-smokers reporting no history of cardiopulmonary disease. All subjects were military divers with at least 5 years’ experience. At the time of the study, they performed a minimum of two training dives per week. All experimental procedures were conducted in line with the declaration of Helsinki. The study protocol was approved by the local ethics committee (Comité de Protection des Personnes-CPP Sud Méditerranée V, ref 160077). Each subject gave written consent before participating in this study.

### Experimental overview

**FIGURE 1:**
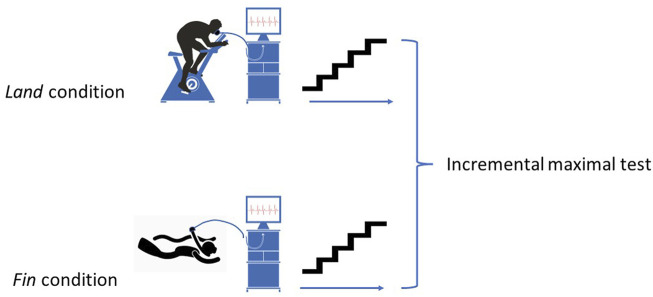
Schematic representations of experimental conditions.

All subjects performed two test exercises on two separates days in no specified order (Figure 1). On day 1, subjects performed a maximal incremental cycle test (*land* condition) in the IRBA Physiology Lab. On day 2, subjects performed a maximal incremental fin swimming test (*fin* condition) in a motorized swimming flume housed within the IRBA Physiology Lab. A rest period of 48–72 h was interposed between tests.

### Spirometry measurements at rest

When taking resting spirometry measurements, in the control condition (*land*), subjects were standing upright in the air. In immersion condition, subjects were positioned upright on their knees, immersed up to the sternal notch (head out of the water immersion) in the pool.

In line with the guidelines of the American Thoracic and European Respiratory Societies [27], forced vital capacity (FVC), forced expiratory volume exhaled in 1 s (FEV_1.0_), peak expiratory Flow (PEF), expiratory reserve volume (ERV), inspiratory reserve volume (IRV), and tidal volume (Vt) were measured. A Cosmed^®^ Quark PFT Ergo device was used (Cosmed^®^, Rome, Italy).

Maximal voluntary ventilation (MVV) was calculated from the FEV_1.0_ using the formula MVV = FEV_1.0_ × 39. Breathing Reserve (BR) i.e., the difference between MVV and the maximum ventilation measured during the exercise test, was determined at peak exercise.

Each subject repeated the spirometry maneuver five times in both *land* and *fin* conditions. The two extreme values for each variable were discarded, and the mean of the remaining three was retained.

### Maximal incremental cycling test (*land* condition)

Participants performed a 10-min standardized 50-W warm-up on a cycle ergometer (E5, COSMED, Rome, Italy). Exercise intensity was then increased stepwise in 25-W increments every minute until volitional exhaustion. Subjects chose a pedaling frequency between 75 and 90 rpm. The selected frequency was maintained throughout the graded exercise test and replicated in all experimental trials.

Heart rate (HR), was monitored with a waterproof heart rate monitor (Polar V800, Helsinki, Finland); tidal volume (Vt), breath frequency (*f*), oxygen uptake ( $\dot{V}$ O_2_) and respiratory exchange ratio (R) were assessed breath by breath using a metabolimeter (PFT Ergo, COSMED, Rome, Italy). Immediately after each test, at the end of the exercise, a blood sample was taken from an ear lobe to analyze partial pressure of oxygen and carbon dioxide (PaO_2_, PaCO_2_) and oxygen saturation (SO_2_) in arterialized blood (i-STAT, Abbott, Chicago United States). One minute later, a second blood sample was taken from the ear lobe to analyze blood lactate [La]_b_ concentrations. Perceived exertion was rated at the end of each step. Body temperature data was systematically specified in the input of system (I-Stat) analysising of blood samples.

$\dot{V}$ O_2max_ was defined as the highest $\dot{V}$ O_2_ value obtained during the test. The $\dot{V}$ O_2max_ was considered valid when, 1) participants rated their perceived exertion above 19 on the Borg scale, 2) - the difference in $\dot{V}$ O_2_ between the last two consecutive workloads was less than 0.10 L min^-1^, 3) - R exceeded 1.10, 4- HR was more than 80% of the age-adjusted estimated maximal HR, and 5- [La]_b_ was higher than 7 mmol.L^-1^ (Smirmaul et al., 2013; Edvardsen et al., 2014).

### Incremental fin swimming on a flume (*fin* condition)

The fin condition was conducted in a motorized swimming flume (Endless Pools, Dilsen-Stokkem, Belgium) housed within the IRBA Physiology Lab. The linearity and the relationship between engine power and water speed were fully calibrated using a water flowmeter.

The same material as for the cycling test was used to measure HR and gas exchanges at mouth-level. To introduce the metabolimeter, the mouth-piece was connected to a snorkel with a low airflow resistance (Dalacqua, Cosmed), as validated by Rodriguez et al. (Rodriguez et al., 2008).

After 10 min of rest, subjects performed a maximal test according to an incremental protocol. After 1 min at a flume speed of 1.0 m s^-1^, the speed was increased by 0.2 m s^-1^ until volitional exhaustion. The $\dot{V}$ O_2max_ recorded was considered valid according to the same criteria as for the land exercise.

### Statistical analysis

Statistical analyses were performed using Prism 6 software (GraphPad Software, La Jolla California United States). Each subject served as his own control. Data distribution was assessed using a Kolmogorov-Smirnov test. To compare the maximum physiological responses from the two incremental tests, a paired Student’s t-test was applied when the data were normally distributed. For non-normally distributed data, a Wilcoxon test was used. The same approach was used to assess lung function at rest in both conditions.

For values obtained at repeated points ( $\dot{V}$ O_2max_ for cycling or surface fin swimming), when the data were normally distributed, one-way repeated-measures analysis of variance was performed (applying the *post hoc* Holm–Sidak test). For non-normally distributed data, comparisons were based on a Friedman’s test and on *post hoc* dichotomous comparisons with a Dunn’s test.

Differences between groups were considered statistically significant at *p* < 0.05. All values are expressed as mean ± SD.

## Results

### Immersion

Lung function was assessed at rest, in both chest-out-of-water and chest immersed (head-out-of-water) conditions. Data are reported in Table 1.

**TABLE 1:** Lung function assessed at rest, on land and during head-out-of-water immersion (Immersion).

	Chest out of water		Chest immersed (head out of the water)	$\frac{chest\;immersed}{chest\;out\;of\;water},\%$	*p*
	Data	% Theoric			
FVC, L	5.42 ± 031	108.75 ± 6.25	4.90 ± 0.39	−9.6 ± 5.1	0.0156
FEV_1_, L.s^−1^	4.38 ± 0.29	102.55 ± 8.92	4.31 ± 0.28	−1.9 ± 0.21	0.345
PEF, L.s^−1^	11.05 ± 0.89	127.28 ± 15.75	11.19 ± 0.72	−1.3 ± 0.1	0.534
Vt, L	5.69 ± 0.43	107.32 ± 2.34	4.95 ± 0.38	−12.8 ± 3.6	0.0002
ERV, L	1.66 ± 0.38	106.54 ± 2.28	0.43 ± 0.35	−76.5 ± 17.9	<0.0001
IRV, L	3.06 ± 0.42	108.85 ± 1.98	3.46 ± 0.32	15.0 ± 11.1	0.0191
MVV, L.min^−1^	170.8 ± 3.5	—	168.3 ± 3.9	−1.6 ± 0.2	0.698

Values correspond to mean ± standard deviation. FVC, forced vital capacity; FEV_1.0_, Forced expiratory volume in 1 s; PEF, peak expiratory flow; Vt, Vital Capacity; ERV, expiratory reserve volume; IRV, inspiratory reserve volume; MVV, maximal voluntary ventilation calculated from pulmonary function data, using the function MVV = FEV_1_ x 39. p, paired Student’s t-test was applied to normally distributed data; for non-normally distributed data, a Wilcoxon test was used.

Immersion was associated with a 74% collapse of ERV (*p* < 0.001), which was not compensated by a 13% increase in IRV (*p* = 0.02). These two immersion situations resulted in small decreases in FVC and Vt (*p* < 0.02 and *p* < 0.001, respectively). Chest immersion induced no further significant alterations to FEV_1.0_, PEF, and MVV. An example of immersion-induced changes in lung function at rest (total lung capacity) is illustrated in the supplementary files (Supplementary Figure S4).

### Incremental tests

**FIGURE 2:**
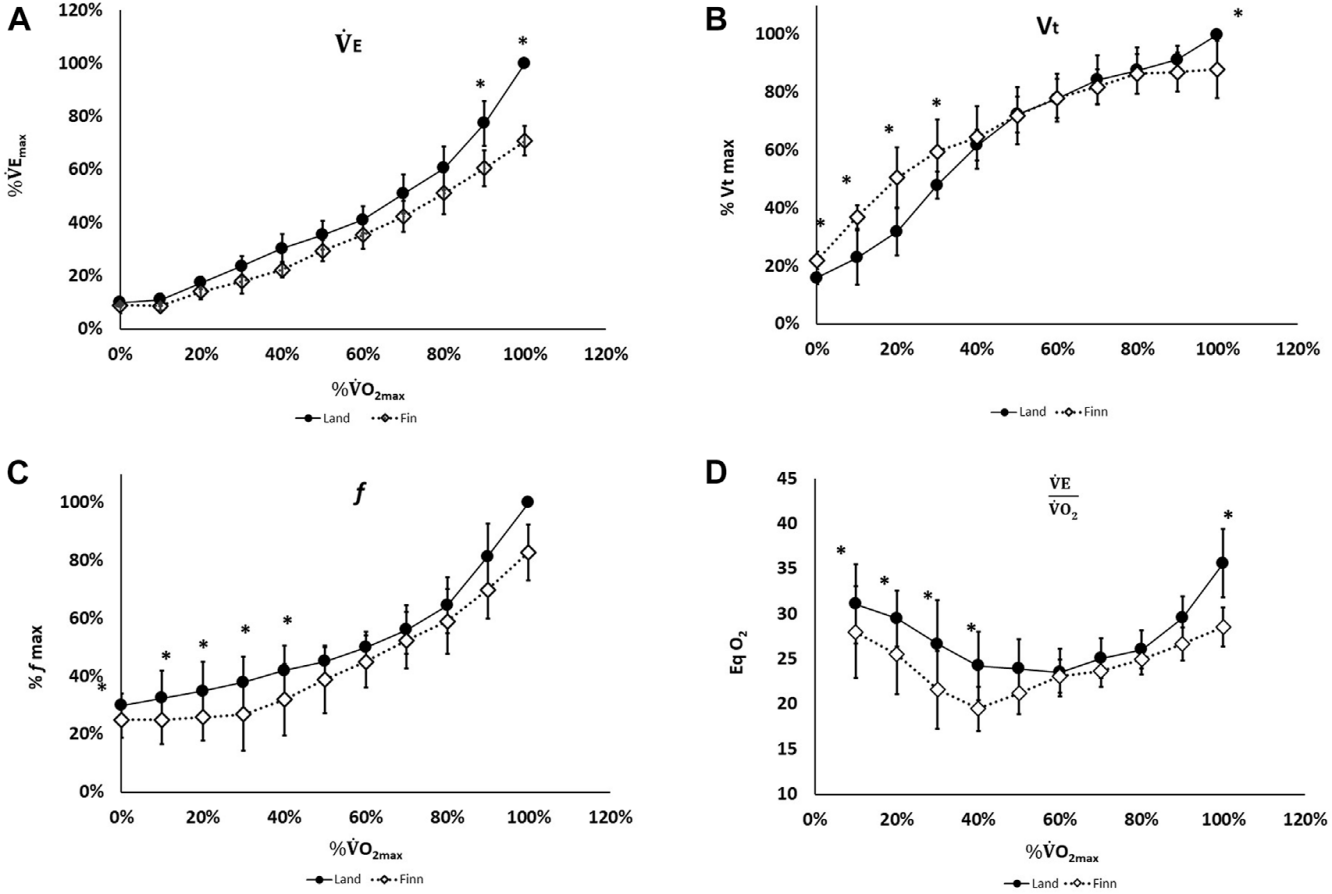
Changes to respiratory variables and heart rate (HR) expressed as a percentage relative to maximal oxygen consumption on land. Variables are expressed as a percentage of the maximum value recorded during the test. $\dot{V}$ *E, pulmonary ventilation; f, breathing frequency; Vt, tidal volume.* Filed symbols: incremental tests in *land* condition; open symbols: incremental tests in *fin* condition. *the difference between the two conditions for the same metabolic load expressed as % $\dot{V}$ *O*_*2max*_ was significant.

To compare the physiological adaptations in response to *land* and *fin* conditions, since no significant difference in $\dot{V}$ O_2max_ was observed between the two conditions, the kinetics of physiological variables were expressed as a percentage of the $\dot{V}$ O_2max_ measured during *cycling*. This is referred to as *land* comparison in the figures presenting changes to ventilatory and HR responses, expressed as function of maximal values during both incremental tests (Figure 2).

No significant difference in $\dot{V}$ E, expressed as % $\dot{V}$ E_max_, between the two conditions was observed as long as the metabolic load remained below 90*%* $\dot{V}$ O_2m*ax*_. $\dot{At\;V}$ O_2m*ax*_ *,* $\dot{V}$ E in the *fin* condition was significantly lower than in the land condition.

At rest and up to an exercise intensity of 40% $\dot{V}$ O_2m*ax*_, *fin* Vt (expressed as %Vt_max_) was significantly higher than *land* Vt; in contrast, *f* (expressed as % *f*_max_) was lower. In the 50%–90% $\dot{V}$ O_2max_ range, *fin* and *land* Vt and *f* values were similar. At $\dot{V}$ O_2max_, *fin* Vt and *fin f* were significantly lower than their *land* counterparts. At rest, and for metabolic loads corresponding to 20%, 30%, 40% and 100% $\dot{V}$ O_2max_, the *fin* $\dot{V}$ E/ $\dot{V}$ O_2_ ratios were significantly lower.

The respective maximal values obtained during incremental cycling and fin swimming tests are reported in Table 2. No significant differences between the two conditions were observed in the maximum values of HR [La]_b_, and R.

**TABLE 2:** Maximum physiological responses observed in cycling (*land* condition) and fin swimming (*fin* condition) incremental tests.

	*land* (cycling)	*fin* (swim flume)	$\frac{land\;incremental}{fin\;incremental},\%$	*p*
$\dot{V}O_{2\;\max},mL.\min^{-1}$	149.9 ± 630	3,701 ± 386	91.8% ± 7%	0.0660
Water flume speed, m.min^−1^	—	51.3 ± 7.7	—	—
$\dot{V}E_{\max},L.\min^{-1}$	149.9 ± 17.6	104.7 ± 8.8	70.9% ± 5.4%	<0.001
BR,L.min^−1^	21.82 ± 2.54	63.12 ± 7.25	289.1% ± 18.2%	<0.001
BR, %VMM	13.25 ± 2.81	38.21 ± 3.36	289.1% ± 18.2%	<0.001
*f*_max_, brpm	45.5 ± 11.2	39.1 ± 7.9	88.3% ± 19.4%	0.002
Vr_max_, L	3.34 ± 0.73	2.77 ± 0.43	84.6% ± 12.5%	0.0353
R_max_	1.11 ± 0.03	1.09 ± 0.01	98,1% ± 3%	0.1762
$\dot{V}E_{\max}/\dot{V}O_{2\;\max}$	36.64 ± 6.70	28.19 ± 5.05	77.8% ± 11.6%	0.0027
[La]_b max_, mmol.L^−1^	10.63 ± 2.28	`0.5` ± 2.59	98.3% ± 0.08%	0.0708
HR_max_, bpm	185.5 ± 12.25	179.1 ± 12.08	96.7% ± 4%	0.128
PaO_2_, mm Hg	100.4 ± 0.8	100.6 ± 0.5	99.8% ± 0.1%	0.254
PaCO_2_, mm Hg	40.2 ± 0.9	40.5 ± 0.7	99.7% ± 0.1%	0.189
SO_2_, %	98.3 ± 1.2	99.1 ± 0.5	99.6% ± 0.2%	0.137

Values presented correspond to mean ± standard deviation. $\dot{V}$ O_2 max_, maximum oxygen uptake; $\dot{V}$ E_max_, maximal minute ventilation; BR, breathing reserve, corresponding to the difference between the calculated maximal voluntary ventilation (MVV) and maximum exercise ventilation ( $\dot{V}$ E_max_) in absolute terms, expressed as a fraction of the MVV; *f*_max_, maximal respiratory frequency; Vt _max_, maximal tidal volume; R _max_, maximal respiratory exchange ratio [La]_b max_, maximal blood lactate concentration; HR _max_, maximal heart rate; PaO_2_, partial pressure of oxygen in arterial blood at the end of exercise; PaCO_2_, partial pressure of carbon dioxide in arterial blood at the end of exercise; SO_2_, oxygen saturation at the end of exercise. p, paired Student’s t-test was applied with normally distributed data; for non-normally distributed data, a Wilcoxon test was used.

**FIGURE 3:**
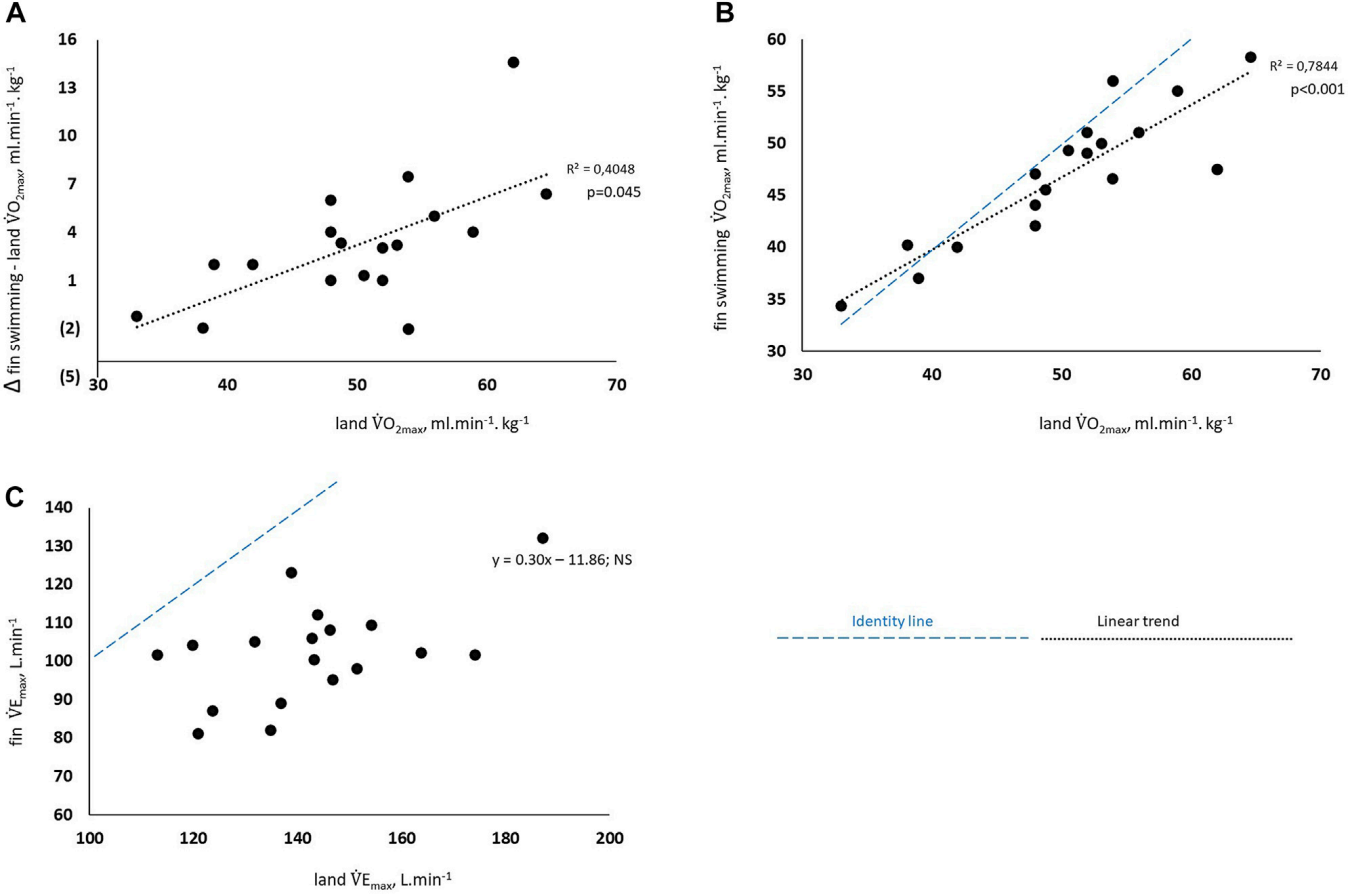
Maximum minute ventilation and maximum oxygen uptake in both land cycling and fin swimming conditions. • **(A)** Changes to differences in maximum oxygen uptake ( $\dot{V}$ O_2max_) measured during incremental tests, expressed relative to land $\dot{V}$ O_2max._ • **(B)** Correlation between the maximum Oxygen uptake ( $\dot{V}$ O_2max_) in the two conditions. • **(C)** Correlation between the maximum pulmonary ventilation ( $\dot{V}$ E_max_) measured in the two conditions. Blue dashed line: identity line; black dashed line: linear trend Statistical significance was based on a Pearson correlation test.

The $\dot{V}$ O_2max_ values recorded in *fin* and *land* conditions were 3,701 ± 39 mL min^-1^ and 4,029 ± 63 mL min^-1^, respectively (*p* = 0.07). The individual *fin*-*land* differences in $\dot{V}$ O_2*max*_ increased with l; $\dot{V}$ O_2max_, (*r* ^2^ = 0.4; *p* = 0.045), Figure 3A. The $\dot{V}$ O_2max_ values for *fin* and *land* were strongly correlated (*r* ^2^ = 0.78 *p* < 0.001), Figure 3B. The *fin* $\dot{V}$ E_*max*_ values were significantly lower than the *l;* $\dot{V}$ E_*max*_ values (*p* < 0.01), and no correlation was observed Figure 3C. These values accounted for both the significantly lower Vt and *f* values (*p* < 0.01 and 0.04, respectively). Consequently, the $\dot{V}$ E_*max*_/ $\dot{V}$ O_2*max*_ ratios were significantly lower in the *fin* condition (*p* < 0.01). In contrast, no significant differences were noted for the maximum values of, PaO_2_, PaCO_2_, and SO_2_ measured in *land* and *fin* conditions.

## Discussion

This mainly descriptive study was designed to investigate an operational military problem: the assessment of metabolic load and of the corresponding variables during a surface fin swimming exercise in well-trained military divers. We first investigated the immersion-specific effects on ventilation to determine whether ventilation would contribute to limiting gas exchanges when performing an incremental surface fin swimming exercise. A second purpose was to determine whether surface fin swimming and land exercise entailed different metabolic loads ( $\dot{V}$ O_2_), specifically whether immersion reduced $\dot{V}$ O_2max_.

Effects of surface immersion on gas exchanges during fin swimming on water surface in trained divers.

In the fin swimming condition, the higher resting Vt and lower resting *f*, *a*ssociated with a lower $\dot{V}$ E/ $\dot{V}$ O_2_ ratio compared to the land condition (Figure 2) confirmed that immersion leads to changes in lung spirometry and reduces lung volume (Thalmann et al., 1979; Taylor et al., 2014). Reduced Vt upon immersion has been repeatedly described (Hong et al., 1969; Craig and Dvorak, 1975; Prefaut et al., 1976; Taylor and Morrison, 1989; Hampson and Dunford, 1997; Taylor and Morrison, 1999). The observations presented here corroborated results presented by Paton and Sand (Paton and Sand, 1947) and Dahlbäck et al. (Dahlback et al., 1978).

### Immersion induced a cephalad displacement of the diaphragm

Immersion led to significant changes in ventilation due to the water pressure exerted on tissues (Choukroun et al., 1989; Christie et al., 1990). Indeed, immersion pushes part of the blood content from the limbs back to the right heart, leading to a tendency for accumulation in the pulmonary circulation and abdominal vascular territory. This type of effect is known as a *blood shift*. From a ventilatory point of view, the *blood shift* leads to pulmonary vascular congestion (Agostoni et al., 1966). The reduced expiratory reserve volume (ERV) associated with head-out-of-water immersion reflected a decrease in residual functional capacity (Paton and Sand, 1947; Jarrett, 1965). By compressing the abdomen, hydrostatic pressure pushes the diaphragm toward the chest, thus reducing chest wall compliance and reducing the pulmonary gas volume. As a consequence, vital capacity is reduced. We recently confirmed observations from 1978 (Dahlback et al., 1978), showing that the immersion-induced changes in lung spirometry volumes are associated with an overall impaired compliance of the respiratory system (Castagna et al., 2021).

During incremental exercise, differences between *land* and *fin* conditions reflect the conflicting effects of immersion and exercise on ventilation. Up to 40% $\dot{V}$ O_2max_, the effects of immersion predominated. From 40% to 80% $\dot{V}$ O_2max_, the effects of immersion and exercise were balanced. Over 90% $\dot{V}$ O_2max_, the $\dot{V}$ E, Vt, and *f* values were significantly lower in the *fin* condition than in the *land* condition*.* This reduction resulted in a 30% lower $\dot{V}$ E_max_.

However, the decreased $\dot{V}$ E_max_ does not mean that ventilation was close to its maximum capacity, since at maximum fin swimming, the ventilatory reserve was greater than in the land condition (37% MVV vs. 12%; *p* < 0.001). Fin swimmers could therefore produce a few cycles of voluntary hyperventilation. Finally, maximum values for the gas exchange variables monitored—PaO_2_, PaCO_2_, and SO_2_—were similar in both conditions.

The $\dot{V}$ E/ $\dot{V}$ O_2_ index reflects ventilatory efficiency, providing an insight into the interaction between pulmonary ventilation, pulmonary perfusion, and cardiac output. The decrease in $\dot{V}$ E_max_/ $\dot{V}$ O_2max_ reported in this study would thus account for an increase in ventilation efficiency as a result of immersion.

This enhanced ventilation efficiency during immersion, could mainly be explained by better lung perfusion. Indeed, the blood shift induced by immersion generated a rise in pulmonary perfusion due to the increase in right ventricle preloading and slight pulmonary vascular congestion.

Altogether the data presented suggest that metabolic gas exchanges are more efficient during maximal fin swimming than in land conditions, due to improved ventilation efficiency upon immersion. The improvement was mainly related to enhanced lung perfusion. The $lower\;\dot{V}$ E_max_ values thus mainly reflected better pulmonary gas exchanges that reduced the need for a high $\dot{V}$ E. These conclusions contrast with current views.

### Effects of surface immersion on $\dot{V}$ O_2max_ during fin swimming on water surface in trained divers

The *land* values recorded for the military divers studied here were higher than those reported for general male subjects in a similar age range (Wilson and Tanaka, 2000; Trappe et al., 2013). This difference confirms the good training status of our subjects. The *fin* $\dot{V}$ O_2max_ values were also higher than some previously reported $\dot{V}$ O_2max_—2.45 L min^-1^ by Pendergast et al. (Pendergast et al., 2003), 2.23 L min^-1^ by Jammes et al. (Jammes et al., 2009) and 2.49 L min^-1^ by Wylegala et al. (Wylegala J. et al., 2007). However, our results were in line with those reported in other studies involving military divers, such as Donald and Davidson (Donald and Davidson, 1954), who reported $\dot{V}$ O_2_ of 3–4 L min^-1^.

An important feature of this study was that immersion does not reduce the maximum aerobic capacity of trained subjects. The $\dot{V}$ O_2_ measured at exhaustion in *land* and *fin* conditions were similar. The similar HR_max_ [La]_bmax_ *land* and *fin* variables (Mier et al., 2012; Smirmaul et al., 2013; Edvardsen et al., 2014) suggest that subjects had reached their maximum aerobic capacity during fin swimming and could stress their energy metabolism to the same extent as during cycling. Interestingly, the *fin* and *land* $\dot{V}$ O_2_/HR ratios were similar at every relative power (Figure 2). Consequently, HR appears to be a good indicator of relative intensity during surface fin swimming.

The decrease in $\dot{V}$ O_2*max*_ was associated with a *p*-value of 0.07, which could hint at a real trend for reduction of this parameter. However, as the differences in $\dot{V}$ O_2*max*_ were not associated with differences in $\dot{V}$ E_max_, the effects of immersion on $\dot{V}$ E_max_ are independent of its effect on $\dot{V}$ O_2*max*_. On the other hand, the *land*-*fin* differences in $\dot{V}$ O_2max_ were related to $\dot{V}$ E_max_ (*r* ^2^ = 0.40; *p* = 0.045), Figure 3A). This would suggest that fitter populations could demonstrate significantly lower $\dot{V}$ O_2max_ in swimming than in *land* condition.

These results contrast with the data obtained by Jammes et al. (Jammes et al., 2009) who compared cardiovascular responses during incremental exercise on a cycle ergometer to an underwater swimming exercise. Although these authors also reported no significant difference in $\dot{V}$ O_2_ measured at exhaustion, they found maximum HR to be 18% lower. No ventilatory threshold was attained, as indicated by the $\dot{V}E$ / $\dot{V}$ O_2_ ratio. Similarly, Yamaguchi et al. (Yamaguchi et al., 1995) reported decreased $\dot{V}$ E and HR at maximal swimming speeds in some of their subjects. However, it is worth noting that whereas the subjects of the present study performed natural swimming movements by fighting against the current of a swimming flume, the subjects described by Jammes et al. (Jammes et al., 2009) and Yamaguchi et al. (Yamaguchi et al., 1995) were stationary, pushing against vertical handles. This difference in effort exerted may have altered central venous circulation. Indeed, the blood shift induced by immersion generated an increase in pulmonary perfusion as a result of increased right ventricle preloading and vascular pulmonary congestion.

## Perspectives and practical interests of the study

Age (over 50 years) and high blood pressure are factors that favor the occurrence of IPE (Gempp et al., 2011; Gempp et al., 2014). However, despite their young age and good health profiles, IPE have become the first cause of hospitalization for military divers - ahead of decompression sickness. The development of IPE in professional male SCUBA divers was previously confirmed by our team to involve a key role of negative pressure breathing induced by hydrostatic imbalance (Castagna et al., 2018b). As hydrostatic imbalance is now taken into account by manufacturers and the military hierarchy, most IPE developing in military divers occur during surface fin swimming exercises. Accidents mainly involve students training to become military divers, and specifically tend to occur during timed surface fin swimming exercises. During these tests, student divers must cover a distance (between 1,000 and 5,000 m) as quickly as possible at the risk of being eliminated. To pass these tests, the students must perform a high-intensity fin swimming exercise lasting several dozen minutes.

The results of the present study demonstrate that pulmonary ventilation related to military fin swimming is much higher than expected. The relationship between an increase in pulmonary ventilation demand and increased work of breathing (WOB) has now been clearly established (Coulange et al., 2010; Bates et al., 2011; Carter and Koehle, 2011; Castagna et al., 2017; Castagna et al., 2018b). Furthermore, some studies have demonstrated the major role played by a high WOB, even in the absence of hydrostatic imbalance, in the occurrence of this type of accident (Peacher et al., 2015; Moon, 2019). We therefore hypothesize that the respiratory work induced by high-intensity fin swimming is susceptible to induce IPE.

Manufacturers of breathing apparatus are developing systems for divers with the lowest possible ventilatory constraints. In Europe, they can rely on the WOB limit values prescribed by European standards (EN 250 and EN 14143). These standards define the maximum acceptable values of WOB in a range of ventilatory regimes ( $\dot{V}$ E) ranging from 10 to 75 L min^-1^. The range of ventilatory regimes tested is based on old and fragmentary measurements, not on recent comprehensive data. Future work, involving a scuba diving condition - with a scuba breathing apparatus - may show that the ventilatory regime of military divers sometimes exceeds the limits set by the standards. If this possibility is confirmed, it could have consequences for divers’ health

## Limitations

It may seem counter-intuitive that, in trained military divers, surface immersion does not lead to a reduction in pulmonary ventilation. Indeed, as immersion reduces lung capacity it should reduce pulmonary ventilation. A study carried out in non-athletic subjects, with no training for swimming or fin swimming, would therefore be necessary to determine whether the results of this study also apply to subjects without specific training in immersion exercise.

Due to this unexpected observation, pulmonary ventilation related to military fin swimming was much higher than predicted, even exceeding the limits set by the standards with which the manufacturers of diving breathing apparatus must comply. We hypothesized that, in military divers, high pulmonary ventilation was associated with a high WOB, which would favor the occurrence of IPE. Unfortunately, we were unable to confirm this hypothesis. The duration usually prescribed for maximal incremental exercise (8–12 min) is too short for clinical signs of IPE to appear. Moreover, no Ultrasound Lung Comets (ULC) were observed on the lung ultrasounds performed at the end of the immersions. It would be necessary to reproduce this study by measuring the WOB required of each subject breath by breath. To perform these measurements, the operational pneumo-baro tachograph, PBO, has been developed in our laboratory (Castagna et al., 2017; Castagna et al., 2018b; Castagna et al., 2021).

The swimming condition for this study was carried out in a laboratory-generated swim flume allowing the precise assessment of metabolic and ventilatory variables. The results obtained will need to be confirmed through measurements on swimmers in the sea.

The results from this study, involving swimming at the surface of the water, cannot be directly transposed to deep underwater diving. Indeed, the density of the gases breathed, increasing with the depth of immersion, the resistance to the flow of gases in the lungs and the breathing apparatus used strongly reduce the ventilatory aptitudes of divers (Thalmann et al., 1979; Held and Pendergast, 2013). Further studies will be necessary to confirm these observations, in particular by carrying out joint measurements of ventilation and WOB in immersion.

In fin condition, the use of the snorkel made breath-by-breath analysis of exhaled gases impossible. Consequently, values were averaged every 10 s. Therefore, we do not have access to the Pet O2 and Pet CO2 values for the fin swimming exercise, making it impossible to calculate/estimate alveolar PO2 during swimming. This would provide valuable data regarding overall pulmonary gas exchange efficiency (e.g., *via* the AaDO2) to confirm the relative contribution of ventilation-perfusion matching.

We observed no significant differences between the values for arterial oxygen saturation (SO_2_), arterial partial pressure of oxygen (PaO_2_), and alveolar partial pressure of oxygen (PACO_2_) measured at rest and immediately after the end of exercise in either condition (land vs fin). Using the equation proposed by Elliot et al. (Elliott et al., 2014), we estimated the values of alveolar partial pressure of carbon dioxygen (PACO_2_). Secondarily, we determined values for the alveolar - arterial difference in partial pressure of oxygen (A-aDO_2_), which is a measure of efficiency for gas exchange. The values of A-a DO_2_ were similar between the two conditions (land vs fin), and no significant difference in the expansion of A-a DO2, induced by physical exercise, was observed whether exercise was performed on land (6.69 ± 0.15 mmHg) or in water (7.27 ± 0.13 mmHg).

Since the peak values of $\dot{V}$ E_max_/ $\dot{V}$ O_2max_ are lower in the fin condition, and since we observed no difference between the values of A-a DO_2_ between the two conditions, this seems to indicate that with a lower pulmonary ventilation rate $\dot{V}$ E, i.e., a lower entry of air and therefore of O_2_ into the alveoli, the O_2_ passes more rapidly to the blood and the red blood cells. This phenomenon can be explained in at least one way: the O_2_ transfer capacity is greater during immersion. This phenomenon has already been observed by Begin et al. (Begin et al., 1976).

## Conclusion

Surface immersion was found not to impair the maximal aerobic power ( $\dot{V}$ O_2max_) in trained divers performing surface fin swimming in a flume. During incremental maximal exercise, the peak aerobic powers measured were similar during land exercise (cycling) and fin swimming. Although reduced by the mechanical effects of immersion, the peak minute ventilation ( $\dot{V}$ E_max_) recorded during surface fin swimming remained higher than previously reported and was sufficient to provide normal pulmonary gas exchanges. We hypothesize that during maximal surface fin swimming, the lower value of $\dot{V}$ E_max_ is not the consequence of an impairment, but rather a response to enhanced pulmonary vascularization.

## Data Availability

The original contributions presented in the study are included in the article/Supplementary Material, further inquiries can be directed to the corresponding author.
