# Supplementary material for: Oxygen uptake ( V˙ O2) and pulmonary ventilation ( V˙ E) during military surface fin swimming in a swimming flume: Effects of surface immersion
Source: Front Physiol. 2023 Mar 6;14:1145204. doi: 10.3389/fphys.2023.1145204 (PMC10025479; doi:10.3389/fphys.2023.1145204)
Supplement: Supplementary file 1 [file DataSheet1.DOCX]

**Supplementary files- figures**

***Figure 1:*** *A diver during the incremental cycling test, breathing through the Cosmed gas analyzer*


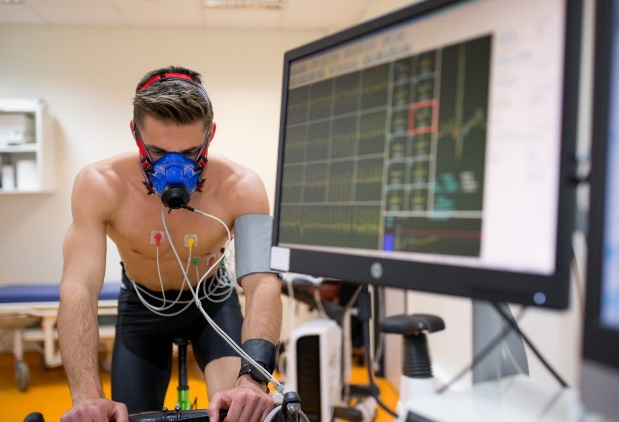


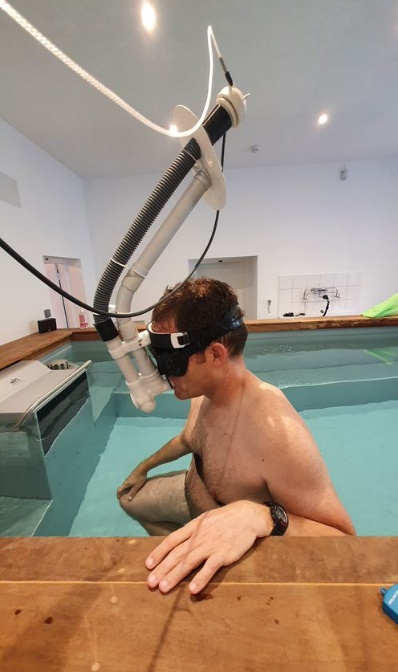
***Figure 2:*** *A diver at rest in the pool, breathing through the Cosmed Snorkel*

***Figure 3:*** *A diver during the fin swimming exercise, breathing through the Cosmed Snorkel*


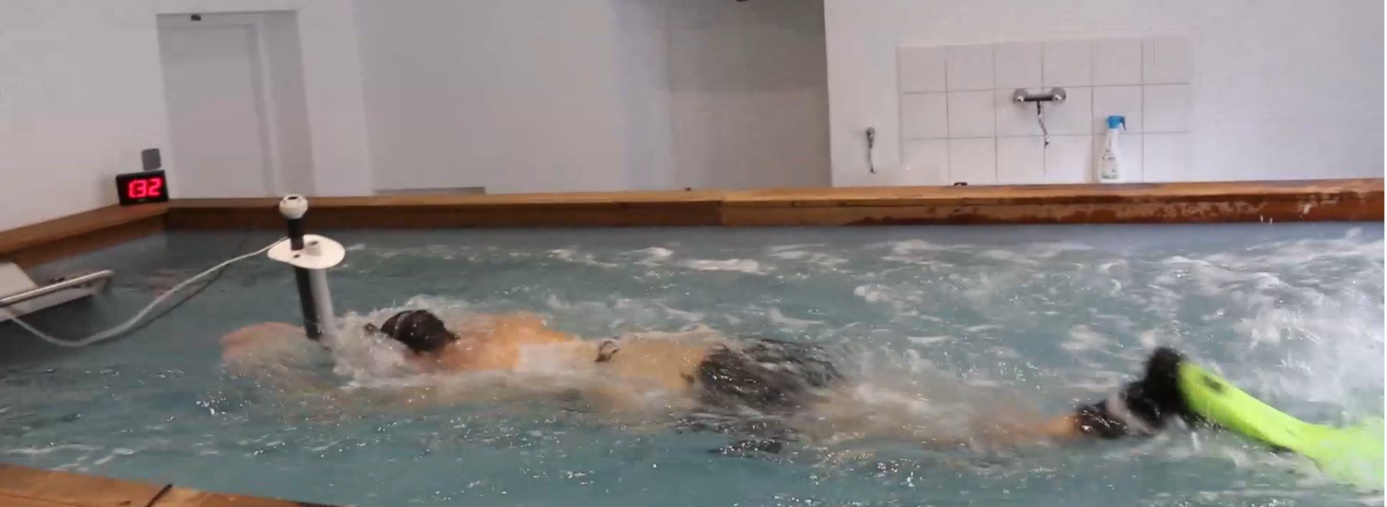


***Figure 4:*** *An example of immersion-induced changes in lung function (total lung capacity) at rest*


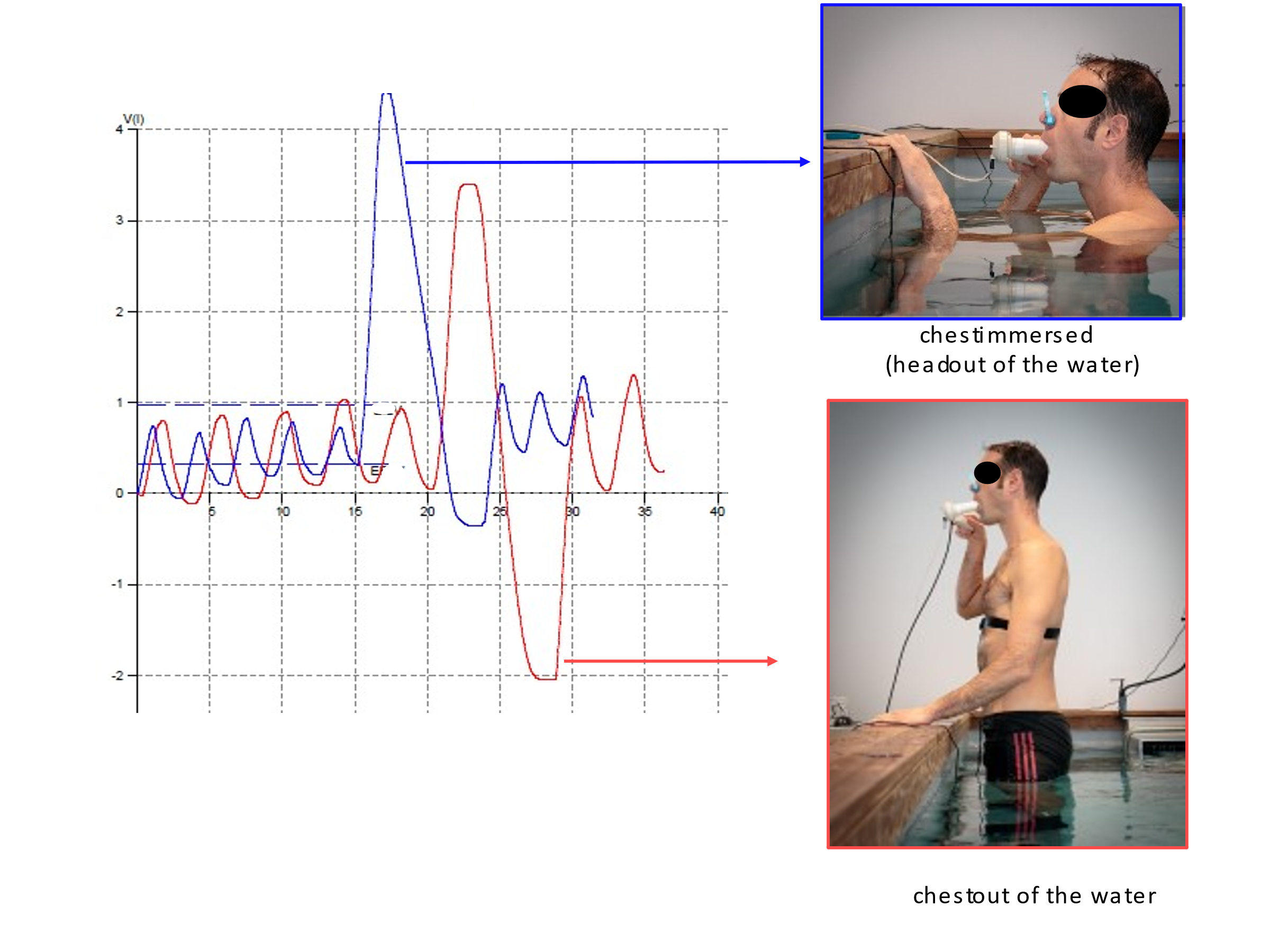


The two curves represent the real values of a subject's TLC. Red curve: chestout-of-water; Blue curve: chest immersed (head-out-of~~-~~water).
